# Supplementary material for: Hematopoietic Cell Transplantation for Systemic Sclerosis—A Review
Source: Cells. 2022 Dec 3;11(23):3912. doi: 10.3390/cells11233912 (PMC9739132; doi:10.3390/cells11233912)
Supplement: Supplementary file 1 [file cells-11-03912-s001.zip › cells-1992347-supplementary.pdf]

cells
MDPI

Supplementary Material

Effects of HCT on multiple manifestations/aspects of SSc

Skin Tightness

Modified Rodnan Skin Score (mRSS) has been used to quantify the degree of skin tightness[1], and assigns values of 0-3 (with a higher score reflecting worse disease) to 17 different body parts, with a maximum score of 51. Faster improvement of mRSS or mRSS improvement to a greater degree after HCT (than measured or expected with conventional therapy) has been documented in all 3 randomized studies[2–4] and multiple non-randomized studies (Figure 1)[5–12]. Collagen density in skin biopsies improves after HCT[7,13]. In one study, the decline of mRSS after HCT correlated with histological assessment of collagen density[7]. It should be pointed out that mRSS usually declines over time even without any immunomodulatory therapy. The rate of decline appears similar in patients without therapy compared to patients treated conventionally with methotrexate, mycophenolate, or cyclophosphamide[14]. However, the mRSS decline is significantly faster after HCT.

Lung Disease

Pulmonary involvement, including interstitial lung disease (ILD) and pulmonary arterial hypertension (PAH), is a major cause of morbidity and mortality in patients with SSc[15]. The prevalence of lung involvement is estimated between 40-75% with pulmonary function tests[16]. Furthermore, ILD occurs more often in the dcSSc versus lcSSc phenotype[17].

ILD has been evaluated using measures of FVC and DLCO, which were found to stabilize or mildly improve after HCT versus decline after conventional therapy (Figure 2) in both randomized [2–4] and non-randomized studies[5,12,18]. Quantitative chest computed tomography (CT) analysis demonstrated improvements in lung volume with reductions in lung density and high attenuation values, reflecting improvement in SSc-mediated ILD after HCT. However, there remains a paucity of robust data to suggest there is improvement in fibrosis after HCT [19]. When compared to cyclophosphamide in a retrospective observational study, HCT decreased (CT)-derived ILD score by 5.1% vs. 1.0% in controls[20]. This was driven largely by improvement in extent of ground glass opacities, which are be considered markers of inflammation (not fibrosis). Another study of CT texture analysis showed improved texture at 6 months in both responders and non-responders, but only responders had sustained improvements at 12 months[21]. Responders were defined as those achieving stabilization or improvement in FVC >10% and DLCO >10%. Similar results have been reported in other CT-based studies[22–25].

The impact of HCT upon PAH has not been studied. This is largely due to exclusion of patients with moderate-severe PAH from HCT trials. In the ASTIS trial, 10 patients (6.6%) had mild-to-moderate PAH at baseline, with more severe patients (those with mean pulmonary artery pressure > 50 mmHg) excluded[3]. Five of the 10 patients with PAH died (2 in the HCT arm and 3 in the control arm), highlighting the considerable risk of HCT and immunomodulation in these patients. However, it was noted in the SCOT trial[4] (which excluded patients with any degree of PAH) that incident PAH developed

in 0/36 patients in the HCT arm vs 5/39 patients in the control arm (p=.02), after 72 months of follow-up. This suggests that HCT might prevent PAH development in SSc, however, the effect of HCT on established PAH remains to be studied. As patients with mild-moderate PAH continue to undergo HCT at some centers[25], it will be important to assess the impact on the progression of PAH during follow up.

Cardiac Disease

Similar to PAH, moderate to severe cardiac involvement has been a contraindication to HCT. Thus, it is unknown whether cardiac disease can improve after HCT. In the SCOT study, clinical heart failure developed in 0/36 patients in the HCT arm vs 4/39 in the control arm by 6 years (p=0.04)[4] and 2/36 vs 7/39 by 11 years[26], suggesting that HCT may prevent or delay heart failure development. However, in the ASTIS study[3], there was no difference in mean left ventricular ejection fraction (LVEF) decline from before to 2 years after treatment between the HCT and control patients. Additionally, measures of diastolic function have not been studied, creating another important gap in in the literature, particularly given the high prevalence of diastolic dysfunction and known myocardial fibrotic involvement observed with SSc[27–29]. As patients with mild to moderate heart disease undergo HCT at select centers [24,25], data on their longitudinal outcomes after HCT will be important.

Esophageal/Gastrointestinal Disease

Despite the gastrointestinal (GI) tract being commonly involved in SSc (up to 90% of patients)[30], there is limited data on its response to HCT. A retrospective study[31] found a mild, albeit statistically significant, reduction of gastrointestinal symptoms in HCT recipients compared to conventionally treated patients. In a retrospective study looking at chest CT pre- and post HCT[32], SSc-related esophageal dilation and volume was shown to worsen despite HCT, with more esophageal dilation seen in patients with progression of ILD. This may suggest that different mechanisms underlie esophageal involvement in SSc. However, radiographic findings of esophageal dilation are a surrogate measure of esophageal function and likely represent the end-stages of esophageal dysmotility. As such, our group has embarked on a prospective study of esophageal motility by high resolution manometry before and annually after HCT. Results on the first 21 patients with amotility or hypomotility preHCT suggest that in patients with amotility, the motility does not improve, whereas in patients with hypomotility, the motility does not worsen or mildly improves (Matthew Woo et al, manuscript in preparation), suggesting that HCT earlier in the SSc course might be beneficial.

Renal Disease

Little is known about the effect of HCT on scleroderma kidney disease. In the SCOT trial[4], 0/36 HCT recipients vs 1/39 controls (p=0.32) developed renal crisis during follow up, but conclusions are limited by small numbers. In the ASTIS[3] trial, creatinine clearance worsened from before to 2 years after treatment to a greater degree in HCT recipients than controls. In a retrospective analysis of 90 HCT patients, [12] 3 patients had renal crisis as their first manifestation of relapse after transplantation, and none of the patients had been maintained on angiotensin blockade. Whether angiotensin blockade reduces the risk of renal events after HCT is unknown. Even in the non-HCT setting the benefit of angiotensin blockade is controversial. While angiotensin blockade has shown survival benefit when used for renal crisis treatment, there is insufficient evidence for efficacy of angiotensin blockade for prevention[33]. One prospective cohort study of 87 non-HCT patients with SSc suggested that exposure to angiotensin blockade prior to renal crisis increased the risk of death [34].

Range of Motion, Strength, and Myositis

In a prospective study of patients from Sao Paolo, Brazil[8], improvement after HCT was noted in the range of motion (ROM) in 5 of 6 hand joints evaluated, finger to palm distance, hand grip strength, and DASH (Disabilities of Arm Shoulder and Hand) questionnaire-evaluated hand function in just 6 months posttransplant. Improvements in wrist ROM and in DASH correlated with improvement of QOL. Improvements were also noted in mouth opening and 6-minute walk test (6MWT). Peak oxygen consumption (VO_{2peak}) during exercise has also been noted to improve after HCT[35]. Here, HCT appeared to improve aerobic capacity, which may be due to improvements in lung, skeletal muscle, and/or cardiac function. Patients with DLCO >70% predicted benefitted most with respect to improvements in aerobic capacity (VO_{2peak}), suggesting that HCT earlier in the disease course may provide greater benefit to patients.

Myositis has not been rigorously evaluated in any published study on HCT for SSc. There is one case report of a 49-year-old woman with myositis who was initially wheelchair-bound pretransplant but no longer required her wheelchair as early as 6 months and out to 7 years posttransplant[36]. Our group has performed HCT in two SSc patients with myositis. It improved both clinically and by creatine kinase levels in both patients. Relapse of the myositis occurred within 1 year in the first patient whereas the myositis was in remission for >2 years in the second patient.

Peripheral Neuropathy

Peripheral neuropathy, reported in SSc with a pooled prevalence of 27% vs. 2-8% in the general population, can affect the motor, sensory and autonomic nerve fibres[37]. No published information on the effect of HCT is available. We have performed HCT in an SSc patient with trigeminal neuralgia, which gradually abated over 5 years posttransplant.

Vasculopathy

Nailfold capillaroscopy (NFC) is considered the gold standard for the assessment of SSc vasculopathy, which usually progresses with conventional therapy over time and has been considered irreversible[38]. However, HCT has been shown to improve capillary patterns[39]. However, a study of skin biopsies showed that vessel density did not significantly change after HCT, despite collagen density and mRSS improvements[40], suggesting that the effect of HCT on SSc vasculopathy may be limited and warrants further study.

Raynaud's phenomenon (RP)

The effect of HCT on RP has been recently retrospectively studied[31] as part of the health-associated quality of life assessment in 41 French patients receiving HCT compared to 65 Canadian patients that did not receive HCT. Patients reported RP scores on the Scleroderma Health Assessment Questionnaire (S-HAQ) at baseline and at subsequent annual follow up visits. At 7 years, RP score improved compared to baseline significantly more in HCT recipients than in controls. Interestingly, the difference was only mild and insignificant in the first 3 years.

Calcinosis

One case report[41] described a 49-year-old female whose severe calcinosis regressed dramatically after HCT. The team in Saskatoon, Canada also witnessed a clinical resolution of severe calcinosis in a young male, whose calcinosis abated by one year posttranspalnt) (Mohamed Elemary, personal communication, September 30, 2022).

Pain

Pain in SSc patients is common and has been reported to affect 83%[42] in one large cohort. Joint pain may be the most common type of pain, with estimated frequency of 46-97%[43,44]. Little is known about the effect of HCT on pain. In one retrospective study[31], HCT recipients reported significantly less pain than conventionally treated patients at 7 years whereas there was only a trend toward less pain at 1 year.

Quality of Life (QOL)

It has been previously shown that in SSc, physical health-related QOL is on average 1.5 standard deviations below that of the general population, which is comparable to other chronic conditions including various cardiopulmonary diseases, diabetes, and depression[45]. Conventional therapies have little to no effect on QOL: When 326 patients treated with cyclophosphamide, methotrexate, MMF, or no immunosuppression were retrospectively compared in the European Scleroderma Observational study, Health Assessment Questionnaire Disability Index (HAQ-DI) scores were similar[14]. In contrast, all 3 randomized studies of HCT vs conventional therapy have shown significantly better QOL after HCT. This was also documented in a recently published systematic review[46], in which patients had better HAQ-DI and SF-36 physical component scores after compared to before HCT. This was also documented in a recent retrospective comparison of HCT recipients with conventionally treated patients[31]. With regards to mental scores, both ASTIS[3] and SCOT[4] reported only modest and statistically insignificant improvements, while ASSIST reported significant improvement in comparison to the cyclophosphamide control group, where deterioration in mental quality of life was observed[2].

Supplementary Table S1. Prospective randomized studies and one important retrospective study of non-HCT therapies vs placebo/another control.

Study	Study Design	Patients (n)	Primary End Point Results	Secondary End Points Results
**Cyclophosphamide (CYC)**				
Tashkin et al. 2006[47]<br><br>Taskin et al. 2007[48]	- Double blind, randomized, placebo-controlled. CYC (2mg/kg/day orally) vs. placebo<br><br>- Follow up study (2007) compared outcomes at 24 months.	158 patients at 13 clinical centers	**- FVC dropped from baseline to 12 months by 1.0% in CYC group vs 2.6% in the placebo group. This was statistically significant when analyzed as mean absolute difference.**<br>**- At 24 months (off CYC since month 12), no significant difference between CYC and placebo groups.**	- Mild but statistically significant greater baseline to 12 mo improvements in skin thickness score and HAQ-DI (from 0.94 to 0.84 after CYC vs from 0.70 to 0.86 after placebo) but not in SF36
Hoyles et al. 2006[49]	- Double blind, randomized, placebo-controlled.<br>- CYC (600mg/m^2 i.v. monthly) + oral azathioprine (2.5mg/kg/day) + low dose oral prednisolone vs. placebo	45 patients from 5 clinical centers	**- No significant difference in FVC was found at 1 year between CYC+Azathioprine+Predisone vs. placebo**	- TLC, DLCO and FEV1 showed no significant difference between CYC vs. placebo
**Mycophenolate Mofetil (MMF)**				
Tashkin et al. 2016[50]	- Double blind, randomized, MMF vs. CYC<br>- MMF (<3mg/daily) vs. CYC (≤2mg/daily)	142 patients; MMF (69), CYC (73)	**- FVC improved from baseline to 2 y in both arms – by 2.9% in CYC and 2.2% in MMF group. The FVC improved in both arms in year 1 and then stabilized.**	- mRSS dropped from baseline to 2 y by 5.3 points in CYC vs 4.9 points in MMF group (not significant)
**Methotrexate (MTX)**				
Van den Hoogen et al. 1996[51]	- Double blind, randomized, MTX vs. placebo	29 patients; MTX (17), placebo (12)	**- More responders by a composite score in MTX arm (53%) than placebo arm (10%) at 24 weeks**	- Creatinine clearance and skin score better at 24 mo after MTX than placebo (borderline significance).

Pope et al. 2001[52]	- Double blind, randomized, placebo-controlled.<br>- MTX orally vs. placebo	71 patients from 8 clinical centers	**- Trend towards faster improvement of mRSS after 12 months of MTX (from 28 to 21) vs placebo (from 27 to 26) (p=.17).**	- Greater improvement of MD visual analog scale (0-10) after 12 months of MTX (from 5.1 to 4.2) vs placebo (from 5.8 to 5.5).
European Scleroderma Observational Study (ESOS) on **CYC vs MMF vs MTX vs No DMARD** - retrospective				
Herrick et al. 2017[14]	- Prospective, non-randomized, observational cohort study<br>- Compared CYC, MMF, MTX, and no DMARD	326 patients from 50 clinical centers; CYC (87), MMF (118), MTX (65), None (56)	**- No difference among the four groups in baseline to 1 or 2 y change of mRSS, FVC, DLCO, or HAQ-DI**	- No significant differences in 2 y survival among groups, however lowest survival in no immunosuppressant group (84%) vs MMF/CYC/MTX groups (88-94%)
**Rituximab (RTX)**				
Daoussis et al. 2010[53]	- Randomized cohort study	14 patients, RTX (8) and control (6, conventional therapy)	**- Significant increase in FVC in RTX arm at 12 months (+10%) vs deterioration (-5%) in control arm**	- Significant increase in DLCO in RTX arm at 12 months (+19.46%) vs. deterioration in control arm (-7.5%)<br>- mRSS scores improved significantly in RTX arm (-5.13)
Sircar et al. 2018[54]	- Prospective, randomized, open-label, parallel group trial<br>- Compared RTX (2g) vs CYC (500mg/m^2)	60 patients, CYC (30) and RTX (30)	**- FVC improved at 6 mo (compared to baseline) in RTX arm (+6%, P=.002) vs. declined in CYC arm (-1%, P=.496)**	- mRSS improved (at 6 mo compared to baseline) in both arms, but possibly to a greater degree in RTX arm (-9.67, P<.001) vs. CYC arm (-5.5, P<.001)
**Tocilizumab (TCZ)**				
Khanna et al. 2016[55]	- Double blind, randomized, placebo-controlled<br>- TCZ 162mg vs. placebo	87 patients from 35 clinical centers.	**- Change in mRSS from baseline to 2 y not significantly different between TCZ and placebo arms**	- Less decline in FVC in TCZ vs. placebo arms at 48 weeks<br>- No difference in change of disability, fatigue, itching, or clinician global disease severity

Khanna et al. 2020[56] Roofeh et al. 2021[57]	- Double blind, randomized, placebo-controlled<br>- TCZ 162mg vs. placebo<br>- Roofeh et al. completed post-hoc analysis on same patients	210 patients from 75 sites; Tocilizumab (104) vs. placebo (106)	**- Change in mRSS from baseline to 4 y not significantly different between TCZ and placebo arms**	- No difference in change of HAQ-DI or patient/physician-global visual analogue scale<br>- In post-hoc analysis, FVC decline better with TCZ (-0.1%) vs placebo (-6.3%)
**Nintedanib**				
Distler et al. 2019[58]	- Double blind, randomized, placebo-controlled<br>- Nintedanib (300mg) vs. placebo	576 patients from 32 centers; Nintedanib (288) vs. placebo (288)	**- Adjusted annual rate of FVC decline lower in nintedanib arm (-52 ml/year) vs. placebo (-93 ml/year) at 12 months**	- No difference in mRSS and St. George's Respiratory Questionnaire at 12 months between ninetedanib vs. placebo at 12 months
**Abatacept**				
Khanna et al. 2020[59]	- Double blind, randomized, placebo-controlled<br>- Abatacept (125mg) vs. placebo	88 patients from 22 centers; Abatacept (44), placebo (44)	**- No significant change in mRSS in Abatacept vs placebo arm at 12 months**	- HAQ-DI improved in Abatacept arm (-0.17 points) vs. worsened in placebo (+0.11 points)
**Riociguat**				
Khanna et al. 2020[60]	- Double blind, randomized, placebo-controlled<br>- Riociguat (1.5-7.5mg) vs. placebo	121 patients; Riociguat (60), placebo (61)	**- No significant change in mRSS in Riociguat vs placebo arm**	

Abbreviations: Cyclophosphamide (CYC), Forced Vital Capacity (FVC), Health Assessment Questionnaire Disability Index (HAQ-DI), Short Form 36 Health Survey (SF36), Total Lung Capacity (TLC), Diffusion Capactiy of Carbon Dixode (DLCO), Forced Expiratory Volume in 1 second (FEV1), Mycophenolate Mofetil (MMF), modified Rodnan Skin Score (mRSS) Methotrexate (MTX), Rituximab (RTX), Tocilizumab (TCZ), Disease-modifying anti-rheumatic drug (DMARD).

Supplementary Table S2. Prospective randomized studies and one important retrospective study of autologous HCT vs control.

Trial:	ASSIST[2]	ASTIS[3]	SCOT[4]	Del Papa et al.[6]
**Trial Basics**				
Type of Trial	Prospective, Randomized (1:1), Phase II, open label, with cross-over to HSCT allowed at 12 months	Prospective, Randomized (1:1), Phase III, open label	Prospective, Randomized (1:1), Phase II, open label	Retrospective, Case:Control design (1:2)
Centers	Single center (Chicago)	29 centers in 10 European countries	26 sites including 8 HCT centers in USA/Canada	Single centre (Milano)
Comparison	HCT vs 6 mo i.v. Cyc	HCT vs 12 mo i.v. Cyc	HCT vs 12 mo i.v. Cyc	HCT vs conventional Rx (mostly Cyc)
Time of Accrual/Randomization	2006 – 2009	2001 – 2009	2005 – 2011	2003 – 2011
**Patient Population**				
Number of Patients	10 HCT vs 9 Cyc	79 HCT vs 77 Cyc	33 HCT vs 34 Cyc (received allocated Rx)	18 HCT vs 36 Ctrl
Non-white race	30% HCT vs 11% Cyc	21% HCT vs 20% Cyc	19% HCT vs 21% Cyc	Not reported
Smokers (ever)	Not reported	52% HCT vs 56% Cyc	39% HCT vs 26% Cyc	Not reported
mRSS baseline (mean)	28 HCT vs 19 Cyc	25 HCT vs 26 Cyc	29 HCT vs 31 Cyc	20 HCT vs 20 Ctrl
LVEF baseline (mean)	Not reported	66% HCT vs 66% Cyc	61% HCT vs 60% Cyc	65% HCTvs62% C
PAH (% of patients)	0% HCT vs 0% Cyc	5% HCT vs 8% Ccy	0% HCT vs 0% Cyc	0% HCT vs 0% C
FVC (mean)	62% HCT vs 67% Cyc	82% HCT vs 81% Cyc	75% HCT vs 74% Cyc	Not reported
DLCO (mean)	58% HCT vs 75% CYC	59% HCT vs 58% CYC	54% HCT vs 53% in CYC	68% HCT vs 67% control
Age (mean or median, years)	45 HCT vs 44 Cyc	44 HCT vs 43 Cyc	45 HCT vs 47 Cyc	41 HCT vs 44 Ctrl
Disease duration (median, years)	1.1 HCT vs 1.5 Cyc	1.4 HCT vs 1.6 Cyc	2.1 HCT vs 2.4 Cyc	2.0 HCT vs 2.0 C
Skin only disease	0%	10%	0%	Not reported
Cyclophosphamide pre-trial (% patients)	10% HCT vs 33% Cyc	22% HCT vs 22% Cyc	22% HCT vs 44% Cyc	22% HCT, control not given

Any DMARD pre-trial (% patients)	Not reported, probably 100% in both arms	Not reported	72% HCT vs 64% Cyc<br>within 6 mo before randomization	100% HCT, control not given
BMI (kg/m^2, mean)	Not reported	25 HCT vs 24 CYC	25 HCT vs 26 CYC	Not reported
Inclusion Criteria	<60 years,<br>Disease duration ≤ 4 y,<br>dcSSc,<br>mRSS ≥ 15,<br>Internal organ involvement	18-65 years,<br>Disease duration ≤ 4 y,<br>dcSSc,<br>mRSS ≥ 15	18-69 years,<br>Disease duration ≤ 4 y,<br>dcSSc,<br>mRSS ≥ 16,<br>Internal organ involvement	No age limit,<br>Disease duration ≤4 years,<br>dcSSc,<br>mRSS ≥ 14,<br>ESSG ≥ 3
Exclusion Criteria	PAPm >25 mmHg<br>PAPsys >40 mmHg<br>LVEF <40%<br>Creatinine>177 µmol/L<br>>6 i.v. Cyc cycles	PAPm >50 mmHg<br>LVEF <45%<br>Creatinine clearance <40 mL/min<br>Cumulative i.v. Cyc dose >5 g or<br>Cumulative oral Cyc dose >3 g	PAPm >30 mmHg<br>LVEF <50%<br>FVC <45% predicted<br>DLCO <40% predicted<br>CrCl <40 mL/min<br>Cumulative Cyc dose >3 g/m^2, >6 i.v. courses, or oral Cyc >4 months	PAPm >25mmHg<br>LVEF <45%<br>DLCO <50% pred<br>Prior renal crisis
**Treatment**				
Mobilization of hematopoietic cells	Cy 2 g/m^2 + GCSF	Cy 4 g/m^2 + GCSF	GCSF	Cy 4 g/m^2 + GCSF
CD34 cell Selection	No	Yes	Yes	Yes
Conditioning Regimen	Cyc 200 mg/kg<br>Rabbit ATG 6.5 mg/kg	Cyc 200mg/kg<br>Rabbit ATG 7.5 mg/kg	Cyc 120 mg/kg<br>TBI 8 Gy, except lungs & kidneys 2 Gy<br>Horse ATG 90 mg/kg	Cyc 200 mg/kg<br>Rabbit ATG<br>7.5 mg/kg
G-CSF post-HCT	10 ug/kg/d from day 5 until engraftment	Not reported	5 ug/kg/d from day 5 until engraftment	Not reported
Prednisone post-HCT	Not reported	Not reported	0.5 mg/kg day 6-21, then taper	Not reported
ACE inhibitor post-HCT	Lisinopril 2.5-10mg/day, no timing details	Recommended, no specific details	Lisinopril 10-20 mg/day from day -5 to day +60	Not reported
Valacyclovir post-HCT	Not reported	Not reported	Until 1 year	Not reported
TMP/SMX post-HCT	Until 6 months	Not reported	Until 1 year	Not reported
Fluconazole post-HCT	Until 6 months	Not reported	Until day 75	Yes, no details
EBV reactivation monitoring	Not reported	Yes, no exact duration of monitoring reported	Yes, until 6 months	Not reported

CMV reactivation monitoring	Not reported	Not reported	Yes, until 12 months post-HCT	Not reported
Vaccinations	Not reported	Not reported	Yes*	Not reported
**Results**				
Follow-up	1.0 y min, 2.6 y mean	5.8 years median	4.5-11.0 years min	5 years min
**Treatment-Related Mortality (TRM)**	**0% HCT vs 0% Cyc at 1 y**	**10% HCT vs 0% Cyc at 1 y**	**3% HCT vs 0% Cyc at 4.5 y, 6% HCT vs 0% Cyc at 6 y**	6% HCT at 1 y, not reported for controls
**Overall Survival**	**100%HCTvs100%Cyc at 1 y**, at a later time could not be evaluated due to crossover design	**86% HCT vs 76% Cyc at 4 y** (P=.002)	**91% HCT vs 76% Cyc at 4.5 y** (P=.02), **88% HCT vs 53% Cyc at 11 y** (P=.01)**	**~90% HCT vs ~45% control at 5 y (P=.002)**
Event-free Survival (EFS)*** or Progression-free Survival (PFS)	NR. PFS appears to be 100% HCT vs 11% Cyc at 1 y, 80% HCT at 2.6 y (NE for Cyc due to crossover)	EFS: 81% HCT vs 74% Cyc at 4 y (P=.006)	EFS: 79% HCT vs 50% Cyc at 4.5 y (P=.02), 74% HCT vs 47% Cyc at 6 y (P=.03)	Not reported
**Disease Progression**	**0% HCT vs 89% Cyc at 1 y,** 20% HCT arm at 2.6 y (Cyc arm not evaluable due to crossover)	Not reported	**9% HCT vs 44% Cyc at 4.5 y** (P=.001), **14% HCT vs 56% Cyc at 11 y** (P value NR)**	NR. **SSc-related mortality was 6% in HCT vs 61% control arm at 5 y** (P<.0005)
**mRSS (absolute change compared to before treatment)******	**Improvement in HCT (-13 points) vs worsening in Cyc (+3 points) at 1 y** (P=.0004)	**Improvement in both arms, but greater in HCT (-20 points) vs Cyc (-9 points) at 2 y** (P<.001)	**Improvement in both arms, but greater in HCT than Cyc at 4.5 y** (P=0.02)*****	**Prob of mRSS falling under 14 points greater in HCT (~100%) vs Ctrl (~70%) at 5 y** (P<.001)
**FVC (absolute change of % predicted compared to before treatment)**	**Improvement in HCT (+12%) vs worsening in Cyc (-6%) at 1 y** (P=.004)	**Improvement in HCT (+6%) vs worsening in Cyc (-3%) at 2 y** (P=.004)	**Improvement in HCT (+4%) vs worsening in Cyc (-14%) at 4.5 y** (P=.005)*****	Not reported
**DLCO (absolute change of % predicted compared to before treatment)**	**Trend toward improvement in HCT (+11%) vs worsening in Cyc (-1%) at 1 y** (P=.34)	**Worsening in both arms to a similar degree, i.e., -5% HCT vs -4% Cyc at 2 y** (P=.84)	**Worsening in both arms, but to a lesser degree in HCT (-4%) vs CYC (-10%) at 4.5 y** (P=.02)*****	**Prob of DLCO falling to <50% ↓ in HCT (~20%) vs Ctrl (~92%) at 5 y** (P=.001)

**Quality of Life (absolute change compared to before treatment)**	**SF36-Physicial: Improvement in HCT (+20 points) vs worsening in Cyc (-6 points)**<br>**at 1 y** (P value for difference between arms not given, but the improvement in HCT arm by 20 points was significant at P=.007)<br><br>SF36-Mental: Improvement in HCT (+12 points) vs worsening in Cyc (-14 points)<br>at 1 y (P value for difference between arms not given, but the worsening in Cyc arm by 14 points was significant at P=.04)	HAQ-DI: Improvement in both arms but greater in HCT (-0.58 points) vs Cyc (-0.19)<br>at 2 y (P=.02)<br><br>**SF36-Physical: Improvement in both arms but greater in HCT (+10 points) vs Cyc (+4 points)**<br>**at 2 y** (P=.01)<br><br>SF36-Mental: Improvement in both arms to a similar degree (+3 points in HCT vs +3 points in Cyc)<br>at 2 y (P=.91)	HAQ-DI: Improvement in HCT (-0.65 points) vs worsening in Cyc (+0.26 points)<br>at 4.5 y (P=.035)*****<br><br>**SF36-Physical: Improvement in both arms but greater in HCT (+15 points) vs Cyc (+2 points)**<br>**at 4.5 y** (P<.001)*****<br><br>SF36-Mental: Trend toward improvement in HCT (+5 points) vs CYC (-2 points)<br>at 4.5 y (P=.11)*****	Not reported

* Vaccinations include heptavalent pneumococcal conjugate vaccine (PCV7), diphtheria, tetanus, haemophilus influenza type b conjugate, hepatitis B, influenza, inactivated polio, measles/mumps/rubella (MMR)
** Sullivan KM et al: Myeloablative autologous hematopoietic stem cell transplantation for severe scleroderma: Long-term outcomes 6-11 years after entry on a randomized study comparing transplantation and cyclophosphamide. American College of Rheumatology 2018 annual meeting, Abstract No. 1820.
*** Event means death or non-fatal heart/lung/kidney failure
**** 0 is normal/no disease, 51 is the maximum degree of skin thickness
***** Mixed effects model as published in Keyes-Elstein L et al: Clinical and molecular findings after autologous stem cell transplantation or cyclophosphamide for scleroderma: Handling missing longitudinal data. Arthritis Care Res (Hoboken), in press (doi: 10.1002/acr.24785).
****** HAQ-DI: 0 indicates best quality of life, whereas 3 indicates worse. SF36-Physical and Mental Component Score: 0 is the worst score, and 100 is the best.

Abbreviations: Cyclophosphamide (Cyc), Mycophenolate Mofetil (MMF), autologous hematopoietic cell transplantation (auto-HCT), left ventricular ejection fraction (LVEF), systemic sclerosis (SSc), modified Rodnan skin score (mRSS), clinical activity score (ESSG), pulmonary artery pressure mean (PAPm), PAPsys (systemic pulmonary artery pressure), forced vital capacity (FVC), diffusion capacity of carbon dioxide (DCLO), anti-thymocyte globulin (ATG), granulocyte colony stimulating factor (G-CSF), Trimethoprim/Sulfamethoxazole (TMP/SMX), Short Form Health Questionnaire 36 (SF36), Creatinine Clearance (CrCl), Total Body Irradiation (TBI), Therapy (Rx), Control(s) (Ctrl or C), Minimum (min), Not evaluable (NE), Not reported (NR), Probability (Prob).

Supplementary Materials References (all references also included in main manuscript, but under a different number)

1. Khanna, D.; Furst, D.E.; Clements, P.J.; Allanore, Y.; Baron, M.; Czirjak, L.; Distler, O.; Foeldvari, I.; Kuwana, M.; Matucci-Cerinic, M.; et al. Standardization of the Modified Rodnan Skin Score for Use in Clinical Trials of Systemic Sclerosis. *J Scleroderma Relat Disord* **2017**, *2*, 11–18, doi:10.5301/JSRD.5000231.
2. Burt, R.K.; Shah, S.J.; Dill, K.; Grant, T.; Gheorghiade, M.; Schroeder, J.; Craig, R.; Hirano, I.; Marshall, K.; Ruderman, E.; et al. Autologous Non-Myeloablative Haemopoietic Stem-Cell Transplantation Compared with Pulse Cyclophosphamide Once per Month for Systemic Sclerosis (ASSIST): An Open-Label, Randomised Phase 2 Trial. *Lancet* **2011**, *378*, 498–506, doi:10.1016/S0140-6736(11)60982-3.
3. van Laar, J.M.; Farge, D.; Sont, J.K.; Naraghi, K.; Marjanovic, Z.; Larghero, J.; Schuerwegh, A.J.; Marijt, E.W.A.; Vonk, M.C.; Schattenberg, A. v.; et al. Autologous Hematopoietic Stem Cell Transplantation vs Intravenous Pulse Cyclophosphamide in Diffuse Cutaneous Systemic Sclerosis: A Randomized Clinical Trial. *JAMA* **2014**, *311*, 2490–2498, doi:10.1001/JAMA.2014.6368.
4. Sullivan, K.M.; Goldmuntz, E.A.; Keyes-Elstein, L.; McSweeney, P.A.; Pinckney, A.; Welch, B.; Mayes, M.D.; Nash, R.A.; Crofford, L.J.; Eggleston, B.; et al. Myeloablative Autologous Stem-Cell Transplantation for Severe Scleroderma. *New England Journal of Medicine* **2018**, *378*, 35–47, doi:10.1056/NEJMOA1703327/SUPPL_FILE/NEJMOA1703327_DISCLOSURES.PDF.
5. Nakamura, H.; Odani, T.; Yasuda, S.; Noguchi, A.; Fujieda, Y.; Kato, M.; Oku, K.; Bohgaki, T.; Sugita, J.; Endo, T.; et al. Autologous Haematopoietic Stem Cell Transplantation for Japanese Patients with Systemic Sclerosis: Long-Term Follow-up on a Phase II Trial and Treatment-Related Fatal Cardiomyopathy. *Mod Rheumatol* **2018**, *28*, 879–884, doi:10.1080/14397595.2017.1416920.
6. del Papa, N.; Onida, F.; Zaccara, E.; Saporiti, G.; Maglione, W.; Tagliaferri, E.; Andracco, R.; Vincenti, D.; Montemurro, T.; Mircoli, L.; et al. Autologous Hematopoietic Stem Cell Transplantation Has Better Outcomes than Conventional Therapies in Patients with Rapidly Progressive Systemic Sclerosis. *Bone Marrow Transplantation 2017 52:1* **2016**, *52*, 53–58, doi:10.1038/bmt.2016.211.
7. Zanin-Silva, D.C.; Santana-Gonçalves, M.; Kawashima-Vasconcelos, M.Y.; Lima-Júnior, J.R.; Dias, J.B.E.; Moraes, D.A.; Covas, D.T.; Malmegrim, K.C.R.; Ramalho, L.; Oliveira, M.C. Autologous Hematopoietic Stem Cell Transplantation Promotes Connective Tissue Remodeling in Systemic Sclerosis Patients. *Arthritis Res Ther* **2022**, *24*, doi:10.1186/S13075-022-02779-W.
8. Costa-Pereira, K.R.; Guimarães, A.L.; Moraes, D.A.; Dias, J.B.E.; Garcia, J.T.; de Oliveira-Cardoso, E.A.; Zombrilli, A.; Leopoldo, V.; Costa, T.M.; Simões, B.P.; et al. Hematopoietic Stem Cell Transplantation Improves Functional Outcomes of Systemic Sclerosis Patients. *J Clin Rheumatol* **2020**, *26*, S131–S138, doi:10.1097/RHU.0000000000001117.
9. Henes, J.; Oliveira, M.C.; Labopin, M.; Badoglio, M.; Scherer, H.U.; Papa, N. del; Daikeler, T.; Schmalzing, M.; Schroers, R.; Martin, T.; et al. Autologous Stem Cell Transplantation for Progressive Systemic Sclerosis: A Prospective Non-Interventional Study from the European Society for Blood and Marrow Transplantation Autoimmune Disease Working Party. *Haematologica* **2021**, *106*, 375, doi:10.3324/HAEMATOL.2019.230128.
10. Shouval, R.; Furie, N.; Raanani, P.; Nagler, A.; Gafter-Gvili, A. Autologous Hematopoietic Stem Cell Transplantation for Systemic Sclerosis: A Systematic Review and Meta-Analysis. *Biol Blood Marrow Transplant* **2018**, *24*, 937–944, doi:10.1016/J.BBMT.2018.01.020.

11. Eyraud, A.; Scouppe, L.; Barnetche, T.; Forcade, E.; Lazaro, E.; Duffau, P.; Richez, C.; Seneschal, J.; Truchetet, M.E. Efficacy and Safety of Autologous Haematopoietic Stem Cell Transplantation in Systemic Sclerosis: A Systematic Review of the Literature. *British Journal of Dermatology* **2018**, *178*, 650–658, doi:10.1111/BJD.15993.
12. Burt, R.K.; Oliveira, M.C.; Shah, S.J.; Moraes, D.A.; Simoes, B.; Gheorghiade, M.; Schroeder, J.; Ruderman, E.; Farge, D.; Chai, Z.J.; et al. Cardiac Involvement and Treatment-Related Mortality after Non-Myeloablative Haemopoietic Stem-Cell Transplantation with Unselected Autologous Peripheral Blood for Patients with Systemic Sclerosis: A Retrospective Analysis. *The Lancet* **2013**, *381*, 1116–1124, doi:10.1016/S0140-6736(12)62114-X.
13. Santana-Gonçalves, M.; Zanin-Silva, D.; Henrique-Neto, Á.; Moraes, D.A.; Kawashima- Vasconcelos, M.Y.; Lima-Júnior, J.R.; Dias, J.B.E.; Bragagnollo, V.; de Azevedo, J.T.C.; Covas, D.T.; et al. Autologous Hematopoietic Stem Cell Transplantation Modifies Specific Aspects of Systemic Sclerosis-Related Microvasculopathy. *Ther Adv Musculoskelet Dis* **2022**, *14*, doi:10.1177/1759720X221084845.
14. Herrick, A.L.; Pan, X.; Peytrignet, S.; Lunt, M.; Hesselstrand, R.; Mouthon, L.; Silman, A.; Brown, E.; Czirják, L.; Distler, J.H.W.; et al. Treatment Outcome in Early Diffuse Cutaneous Systemic Sclerosis: The European Scleroderma Observational Study (ESOS). *Ann Rheum Dis* **2017**, *76*, 1207–1218, doi:10.1136/ANNRHEUMDIS-2016-210503.
15. Steen, V.D.; Conte, C.; Owens, G.R.; Medsger, T.A. Severe Restrictive Lung Disease in Systemic Sclerosis. *Arthritis Rheum* **1994**, *37*, 1283–1289, doi:10.1002/ART.1780370903.
16. Solomon, J.J.; Olson, A.L.; Fischer, A.; Bull, T.; Brown, K.K.; Raghu, G. Scleroderma Lung Disease. *European Respiratory Review* **2013**, *22*, 6–19, doi:10.1183/09059180.00005512.
17. Nihtyanova, S.I.; Schreiber, B.E.; Ong, V.H.; Rosenberg, D.; Moinzadeh, P.; Coghlan, J.G.; Wells, A.U.; Denton, C.P. Prediction of Pulmonary Complications and Long-Term Survival in Systemic Sclerosis. *Arthritis Rheumatol* **2014**, *66*, 1625–1635, doi:10.1002/ART.38390.
18. Henrique-Neto, Á.; Vasconcelos, M.Y.K.; Dias, J.B.E.; de Moraes, D.A.; Gonçalves, M.S.; Zanin-Silva, D.C.; Zucoloto, T.G.; de Oliveira, M. de F.C.; Dotoli, G.M.; Weffort, L.F.; et al. Hematopoietic Stem Cell Transplantation for Systemic Sclerosis: Brazilian Experience. *Advances in Rheumatology* **2021**, *61*, 1–10, doi:10.1186/S42358-021-00166-8/FIGURES/4.
19. Kloth, C.; Maximilian Thaiss, W.; Preibsch, H.; Mark, K.; Kötter, I.; Hetzel, J.; Nikolaou, K.; Henes, J.; Horger, M. Quantitative Chest CT Analysis in Patients with Systemic Sclerosis before and after Autologous Stem Cell Transplantation: Comparison of Results with Those of Pulmonary Function Tests and Clinical Tests. *Rheumatology (Oxford)* **2016**, *55*, 1763–1770, doi:10.1093/RHEUMATOLOGY/KEW259.
20. Ciaffi, J.; van Leeuwen, N.M.; Boonstra, M.; Kroft, L.J.M.; Schouffoer, A.A.; Ninaber, M.K.; Huizinga, T.W.J.; de Vries-Bouwstra, J.K. Evolution of Systemic Sclerosis–Associated Interstitial Lung Disease One Year After Hematopoietic Stem Cell Transplantation or Cyclophosphamide. *Arthritis Care Res (Hoboken)* **2022**, *74*, 433–441, doi:10.1002/ACR.24451/ABSTRACT.
21. Kloth, C.; Henes, J.; Xenitidis, T.; Thaiss, W.M.; Blum, A.C.; Fritz, J.; Nikolaou, K.; Horger, M.; Ioanoviciu, S.D. Chest CT Texture Analysis for Response Assessment in Systemic Sclerosis. *Eur J Radiol* **2018**, *101*, 50–58, doi:10.1016/J.EJRAD.2018.01.024.
22. Wada, D.T.; de Almeida, F.A.; de Moraes, D.A.; Dias, J.B.E.; Baddini-Martinez, J.; Oliveira, M.C.; Koenigkam-Santos, M. Automatic Quantitative Computed Tomography Evaluation of the Lungs in Patients With Systemic Sclerosis Treated With Autologous Stem Cell Transplantation. *J Clin Rheumatol* **2020**, *26*, S158–S164, doi:10.1097/RHU.0000000000001242.
23. Pugnet, G.; Petermann, A.; Collot, S.; Otal, P.; Lansiaux, P.; Maria, A.; Abdallah, N.A.; Lorillon, G.; Resch-Rigon, M.; Marjanovic, Z.; et al. POS0069 LONGITUDINAL CHANGES ON QUANTITATIVE CHEST HIGH-RESOLUTION COMPUTED TOMOGRAPHY IN EARLY DIFFUSE SYSTEMIC SCLEROSIS-RELATED INTERSTITIAL LUNG

DISEASE AFTER AUTOLOGOUS HEMATOPOIETIC STEM CELL TRANSPLANTATION. *Ann Rheum Dis* **2022**, *81*, 251–251, doi:10.1136/ANNRHEUMDIS-2022-EULAR.4608.

24. Henes, J.C.; Koetter, I.; Horger, M.; Schmalzing, M.; Mueller, K.; Eick, C.; Bauer, A.; Vogel, W.; Kanz, L. Autologous Stem Cell Transplantation with Thiotepa-Based Conditioning in Patients with Systemic Sclerosis and Cardiac Manifestations. *Rheumatology (Oxford)* **2014**, *53*, 919–922, doi:10.1093/RHEUMATOLOGY/KET464.
25. Burt, R.K.; Han, X.; Quigley, K.; Arnautovic, I.; Shah, S.J.; Lee, D.C.; Freed, B.H.; Jovanovic, B.; Helenowski, I.B. Cardiac Safe Hematopoietic Stem Cell Transplantation for Systemic Sclerosis with Poor Cardiac Function: A Pilot Safety Study That Decreases Neutropenic Interval to 5 Days. *Bone Marrow Transplantation 2020 56:1* **2020**, *56*, 50–59, doi:10.1038/s41409-020-0978-2.
26. Sullivan, K.; Pinckney, A.; Goldmuntz, E.; Welch, B.; Khanna, D.; Simms, R.; Kafaja, S.; Georges, G.; Storek, J.; Csuka, M.E.; et al. Myeloablative Autologous Hematopoietic Stem Cell Transplantation for Severe Scleroderma: Long-Term Outcomes 6-11 Years after Entry on a Randomized Study Comparing Transplantation and Cyclophosphamide. In Proceedings of the 2018 ACR/ARHP Annual Meeting; 2018.
27. Gotschy, A.; Jordan, S.; Stoeck, C.T.; von Deuster, C.; Peer, T.; Gastl, M.; Vishnevskiy, V.; Wissmann, L.; Dobrota, R.; Mihai, C.; et al. Diffuse Myocardial Fibrosis Precedes Subclinical Functional Myocardial Impairment and Provides Prognostic Information in Systemic Sclerosis. *Eur Heart J Cardiovasc Imaging*, doi:10.1093/EHJCI/JEAC094.
28. Faludi, R.; Költo, G.; Bartos, B.; Csima, G.; Czirják, L.; Komócsi, A. Five-Year Follow-up of Left Ventricular Diastolic Function in Systemic Sclerosis Patients: Determinants of Mortality and Disease Progression. *Semin Arthritis Rheum* **2014**, *44*, 220–227, doi:10.1016/J.SEMARTHRIT.2014.04.001.
29. Hachulla, A.L.; Launay, D.; Gaxotte, V.; de Groote, P.; Lamblin, N.; Devos, P.; Hatron, P.Y.; Beregi, J.P.; Hachulla, E. Extended Report: Cardiac Magnetic Resonance Imaging in Systemic Sclerosis: A Cross-Sectional Observational Study of 52 Patients. *Ann Rheum Dis* **2009**, *68*, 1878, doi:10.1136/ARD.2008.095836.
30. Denaxas, K.; Ladas, S.D.; Karamanolis, G.P. Evaluation and Management of Esophageal Manifestations in Systemic Sclerosis. *Ann Gastroenterol* **2018**, *31*, 165, doi:10.20524/AOG.2018.0228.
31. Maltez, N.; Puyade, M.; Wang, M.; Lansiaux, P.; Marjanovic, Z.; Charles, C.; Steele, R.; Baron, M.; Colmegna, I.; Hudson, M.; et al. Association of Autologous Hematopoietic Stem Cell Transplantation in Systemic Sclerosis With Marked Improvement in Health-Related Quality of Life. *Arthritis Rheumatol* **2021**, *73*, 305–314, doi:10.1002/ART.41519.
32. Pugnet, G.; Petermann, A.; Collot, S.; Otal, P.; Lansiaux, P.; Ait Abdallah, N.; Lorillon, G.; Resche-Rigon, M.; Borel, C.; Marjanovic, Z.; et al. Changes on Chest HRCT in Systemic Sclerosis-Related Interstitial Lung Disease after Autologous Haematopoietic Stem Cell Transplantation. *Rheumatology* **2022**, doi:10.1093/RHEUMATOLOGY/KEAC319.
33. Kowal-Bielecka, O.; Fransen, J.; Avouac, J.; Becker, M.; Kulak, A.; Allanore, Y.; Distler, O.; Clements, P.; Cutolo, M.; Czirjak, L.; et al. Update of EULAR Recommendations for the Treatment of Systemic Sclerosis. *Ann Rheum Dis* **2017**, *76*, 1327–1339, doi:10.1136/ANNRHEUMDIS-2016-209909.
34. Hudson, M.; Baron, M.; Tatibouet, S.; Furst, D.E.; Khanna, D.; Hummers, L.; Hachulla, E.; Medsger, T.; Steen, V.; Alkassab, F.; et al. Exposure to ACE Inhibitors Prior to the Onset of Scleroderma Renal Crisis-Results from the International Scleroderma Renal Crisis Survey. *Semin Arthritis Rheum* **2014**, *43*, 666–672, doi:10.1016/J.SEMARTHRIT.2013.09.008.
35. Gadioli, L.P.; Costa-Pereira, K.; Dias, J.B.E.; Moraes, D.A.; Crescêncio, J.C.; Schwartzmann, P. v; Gallo-Júnior, L.; Schmidt, A.; Oliveira, M.C. Autologous Stem Cell Transplantation Improves Cardiopulmonary Exercise Testing Outcomes in Systemic Sclerosis Patients. *Rheumatology* **2022**, doi:10.1093/RHEUMATOLOGY/KEAC413.
36. Penglase, R.; Englert, H.; Tymms, K.; Carr, A.; Ma, D.; Milliken, S.; Moore, J. Successful Lymphoablative Autologous Haematopoietic Stem Cell Transplantation in Three Cases of Severe Autoimmune Disease despite Reduced Dose

Cyclophosphamide Conditioning—Do We Need 200 Mg/Kg Cyclophosphamide? *Bone Marrow Transplantation 2022 57:7* **2022**, *57*, 1207–1209, doi:10.1038/s41409-022-01696-5.
37. Almehmadi, B.A.; To, F.Z.; Anderson, M.A.; Johnson, S.R. Epidemiology and Treatment of Peripheral Neuropathy in Systemic Sclerosis. *J Rheumatol* **2021**, *48*, 1839–1849, doi:10.3899/JRHEUM.201299.
38. Sulli, A.; Pizzorni, C.; Smith, V.; Zampogna, G.; Ravera, F.; Cutolo, M. Timing of Transition between Capillaroscopic Patterns in Systemic Sclerosis. *Arthritis Rheum* **2012**, *64*, 821–825, doi:10.1002/ART.33463.
39. Aschwanden, M.; Daikeler, T.; Jaeger, K.A.; Thalhammer, C.; Gratwohl, A.; Matucci-Cerinic, M.; Tyndall, A. Rapid Improvement of Nailfold Capillaroscopy after Intense Immunosuppression for Systemic Sclerosis and Mixed Connective Tissue Disease. *Ann Rheum Dis* **2008**, *67*, 1057–1059, doi:10.1136/ARD.2007.082008.
40. Daikeler, T.; Kump, E.; Stern, M.; Hügle, T.; Hij, A.; Haeuserman, P.; Farge, D. Autologous Hematopoietic Stem Cell Transplantation Reverses Skin Fibrosis but Does Not Change Skin Vessel Density in Patients with Systemic Sclerosis. *Pathol Biol (Paris)* **2015**, *63*, 164–168, doi:10.1016/J.PATBIO.2015.07.006.
41. Abdalla, A.; Khalid, M.; Ishaq, Q.; Donnelly, S. Scleroderma-Associated Cutaneous Calcinosis: A Remarkable Regression Post Stem Cell Transplantation. *Clin Rheumatol* **2021**, *40*, 789–790, doi:10.1007/S10067-020-05368-X/FIGURES/1.
42. Schieir, O.; Thombs, B.D.; Hudson, M.; Boivin, J.F.; Steele, R.; Bernatsky, S.; Hanley, J.; Baron, M.; Pope, J.; Markland, J.; et al. Prevalence, Severity, and Clinical Correlates of Pain in Patients with Systemic Sclerosis. *Arthritis Care Res (Hoboken)* **2010**, *62*, 409–417, doi:10.1002/ACR.20108.
43. TUFFANELLI, D.L.; WINKELMANN, R.K. Systemic Scleroderma: A Clinical Study of 727 Cases. *Arch Dermatol* **1961**, *84*, 359–371, doi:10.1001/ARCHDERM.1961.01580150005001.
44. Baron, M.; Lee, P.; Keystone, E.C. The Articular Manifestations of Progressive Systemic Sclerosis (Scleroderma). *Ann Rheum Dis* **1982**, *41*, 147–152, doi:10.1136/ARD.41.2.147.
45. Hudson, M.; Thombs, B.D.; Steele, R.; Panopalis, P.; Newton, E.; Baron, M. Quality of Life in Patients with Systemic Sclerosis Compared to the General Population and Patients with Other Chronic Conditions. *J Rheumatol* **2009**, *36*, 768–772, doi:10.3899/JRHEUM.080281.
46. Puyade, M.; Maltez, N.; Lansiaux, P.; Pugnet, G.; Roblot, P.; Colmegna, I.; Hudson, M.; Farge, D. Health-Related Quality of Life in Systemic Sclerosis before and after Autologous Haematopoietic Stem Cell Transplant-a Systematic Review. *Rheumatology (Oxford)* **2020**, *59*, 779–789, doi:10.1093/RHEUMATOLOGY/KEZ300.
47. Tashkin, D.P.; Elashoff, R.; Clements, P.J.; Goldin, J.; Roth, M.D.; Furst, D.E.; Arriola, E.; Silver, R.; Strange, C.; Bolster, M.; et al. Cyclophosphamide versus Placebo in Scleroderma Lung Disease. *New England Journal of Medicine* **2006**, *354*, 2655–2666, doi:10.1056/NEJMOA055120/SUPPL_FILE/NEJM_TASHKIN_2655SA1.PDF.
48. Tashkin, D.P.; Elashoff, R.; Clements, P.J.; Roth, M.D.; Furst, D.E.; Silver, R.M.; Goldin, J.; Arriola, E.; Strange, C.; Bolster, M.B.; et al. Effects of 1-Year Treatment with Cyclophosphamide on Outcomes at 2 Years in Scleroderma Lung Disease. *Am J Respir Crit Care Med* **2007**, *176*, 1026, doi:10.1164/RCCM.200702-326OC.
49. Hoyles, R.K.; Ellis, R.W.; Wellsbury, J.; Lees, B.; Newlands, P.; Goh, N.S.L.; Roberts, C.; Desai, S.; Herrick, A.L.; McHugh, N.J.; et al. A Multicenter, Prospective, Randomized, Double-Blind, Placebo-Controlled Trial of Corticosteroids and Intravenous Cyclophosphamide Followed by Oral Azathioprine for the Treatment of Pulmonary Fibrosis in Scleroderma. *Arthritis Rheum* **2006**, *54*, 3962–3970, doi:10.1002/ART.22204.
50. Tashkin, D.P.; Roth, M.D.; Clements, P.J.; Furst, D.E.; Khanna, D.; Kleerup, E.C.; Goldin, J.; Arriola, E.; Volkmann, E.R.; Kafaja, S.; et al. Mycophenolate Mofetil versus Oral Cyclophosphamide in Scleroderma-Related Interstitial Lung Disease (SLS II): A Randomised Controlled, Double-Blind, Parallel Group Trial. *Lancet Respir Med* **2016**, *4*, 708–719, doi:10.1016/S2213-2600(16)30152-7.

51. van den Hoogen, F.H.J.; Boerbooms, A.M.T.; Swaak, A.J.G.; Rasker, J.J.; van Lier, H.J.J.; van de Putte, L.B.A. Comparison of Methotrexate with Placebo in the Treatment of Systemic Sclerosis: A 24 Week Randomized Double-Blind Trial, Followed by a 24 Week Observational Trial. *Rheumatology* **1996**, *35*, 364–372, doi:10.1093/RHEUMATOLOGY/35.4.364.
52. Pope, J.; Bellamy, N.; Seibold, J.; Baron, M.; Ellman, M.; Carette, S.; Smith, C.; Chalmers, I.; Hong, P.; O'Hanlon, D.; et al. A Randomized, Controlled Trial of Methotrexate versus Placebo in Early Diffuse Scleroderma. *Arthritis Rheum* **2001**, *44*, 1351–1358, doi:10.1002/art.10088.
53. Daoussis, D.; Melissaropoulos, K.; Sakellaropoulos, G.; Antonopoulos, I.; Markatseli, T.E.; Simopoulou, T.; Georgiou, P.; Andonopoulos, A.P.; Drosos, A.A.; Sakkas, L.; et al. A Multicenter, Open-Label, Comparative Study of B-Cell Depletion Therapy with Rituximab for Systemic Sclerosis-Associated Interstitial Lung Disease. *Semin Arthritis Rheum* **2017**, *46*, 625–631, doi:10.1016/J.SEMARTHRIT.2016.10.003.
54. Sircar, G.; Goswami, R.P.; Sircar, D.; Ghosh, A.; Ghosh, P. Intravenous Cyclophosphamide vs Rituximab for the Treatment of Early Diffuse Scleroderma Lung Disease: Open Label, Randomized, Controlled Trial. *Rheumatology (Oxford)* **2018**, *57*, 2106–2113, doi:10.1093/RHEUMATOLOGY/KEY213.
55. Khanna, D.; Denton, C.P.; Jahreis, A.; van Laar, J.M.; Frech, T.M.; Anderson, M.E.; Baron, M.; Chung, L.; Fierlbeck, G.; Lakshminarayanan, S.; et al. Safety and Efficacy of Subcutaneous Tocilizumab in Adults with Systemic Sclerosis (FaSScinate): A Phase 2, Randomised, Controlled Trial. *Lancet* **2016**, *387*, 2630–2640, doi:10.1016/S0140-6736(16)00232-4.
56. Khanna, D.; Lin, C.J.F.; Furst, D.E.; Goldin, J.; Kim, G.; Kuwana, M.; Allanore, Y.; Matucci-Cerinic, M.; Distler, O.; Shima, Y.; et al. Tocilizumab in Systemic Sclerosis: A Randomised, Double-Blind, Placebo-Controlled, Phase 3 Trial. *Lancet Respir Med* **2020**, *8*, 963–974, doi:10.1016/S2213-2600(20)30318-0.
57. Roofeh, D.; Lin, C.J.F.; Goldin, J.; Kim, G.H.; Furst, D.E.; Denton, C.P.; Huang, S.; Khanna, D. Tocilizumab Prevents Progression of Early Systemic Sclerosis-Associated Interstitial Lung Disease. *Arthritis Rheumatol* **2021**, *73*, 1301–1310, doi:10.1002/ART.41668.
58. Distler, O.; Highland, K.B.; Gahlemann, M.; Azuma, A.; Fischer, A.; Mayes, M.D.; Raghu, G.; Sauter, W.; Girard, M.; Alves, M.; et al. Nintedanib for Systemic Sclerosis–Associated Interstitial Lung Disease. *New England Journal of Medicine* **2019**, *380*, 2518–2528, doi:10.1056/NEJMOA1903076/SUPPL_FILE/NEJMOA1903076_DATA-SHARING.PDF.
59. Khanna, D.; Spino, C.; Johnson, S.; Chung, L.; Whitfield, M.L.; Denton, C.P.; Berrocal, V.; Franks, J.; Mehta, B.; Molitor, J.; et al. Abatacept in Early Diffuse Cutaneous Systemic Sclerosis: Results of a Phase II Investigator-Initiated, Multicenter, Double-Blind, Randomized, Placebo-Controlled Trial. *Arthritis Rheumatol* **2020**, *72*, 125–136, doi:10.1002/ART.41055.
60. Khanna, D.; Allanore, Y.; Denton, C.P.; Kuwana, M.; Matucci-Cerinic, M.; Pope, J.E.; Atsumi, T.; Bečvár, R.; Czirják, L.; Hachulla, E.; et al. Riociguat in Patients with Early Diffuse Cutaneous Systemic Sclerosis (RISE-SSc): Randomised, Double-Blind, Placebo-Controlled Multicentre Trial. *Ann Rheum Dis* **2020**, *79*, 618–625, doi:10.1136/ANNRHEUMDIS-2019-216823.
